# Use of Social Media for Implementing Diagnoses, Consultation, Training, and Case Reporting Among Medical Professionals to Improve Patient Care: Case Study of WeChat Groups Across Health Care Settings

**DOI:** 10.2196/26419

**Published:** 2022-07-29

**Authors:** Lai Sze Tso

**Affiliations:** 1 Global Health & Medical Humanities Initiative Massachusetts Institute of Technology Cambridge, MA United States; 2 Center for Health & Human Development Studies Sun Yat-Sen University Guangzhou China; 3 Institutt for Kulturstudier og Orientalske Språk-Norges Forskningsråd (IKOS-NFR) University of Oslo Oslo Norway; 4 Sociology & Anthropology Gustavus Adolphus College Saint Peter, MN United States

**Keywords:** mHealth, WeChat, implementation research, low-resource settings, innovative medical technologies, digital health, medical education, social media, mobile health technologies, bottom-up approach

## Abstract

**Background:**

Health professionals in low- and middle-resource settings have limited access to up-to-date resources for diagnosing and treating illnesses, training medical staff, reviewing newly disseminated guidelines and publications, and preparing data for international disease reporting. A concomitant difficulty in high-resource settings is the need for continuing education and skills up-training in innovative procedures on unfamiliar social media platforms. These challenges can delay both patient care and epidemiological surveillance efforts. To overcome these challenges, health professionals have adapted WeChat Groups to implement timely, low-cost, and high-quality patient care.

**Objective:**

The primary study aim was to describe the processes taken by medical professionals across their diverse physical and virtual networks in adapting a bottom-up approach to collectively overcome resource shortages. The secondary study aim was to delineate the pathways, procedures, and resource/information sharing implemented by medical professionals using an international publicly available popular social media app (WeChat) to enhance performance of facility-based procedures and protocols for improved patient care.

**Methods:**

In-depth interviews, observations, and digital ethnography of WeChat Groups communications were collected from medical professionals in interconnected networks of health care facilities. Participants’ WeChat Groups usage and observations of their professional functions in interconnected networks were collected from November 2018 to 2019. Qualitative analysis and thematic coding were used to develop constructs and themes in NVivo. Constructs incorporated descriptions for the implementation and uses of WeChat Groups for professional connections, health care procedures, and patient care. Themes supporting the constructs focused on the pathways and venues used by medical professionals to build trust, to establish and solidify online networks, and to identify requests and resource sharing within WeChat Groups.

**Results:**

There were 58 participants (males 36 and females 22) distributed across 24 health care settings spanning geographical networks in south China. Analysis yielded 4 constructs and 11 themes delineating the bottom-up usage of WeChat Groups among clinicians, technicians, nurses, pharmacists, and public health administrators. Participants used WeChat Groups for collectively training hospital staff in complex new procedures, processing timely diagnoses of biological specimens, staying abreast of latest trends and clinical procedures and symptoms, and contributing to case reporting for emergent illnesses and international surveillance reporting. An unexpected strength of implementing clinical, training, and research support on a popular app with international coverage is the added ability to overcome administrative and geographic barriers in resource distribution. This advantage increased a network’s access to WeChat Groups members both working within China and abroad, greatly expanding the scope of shared resources.

**Conclusions:**

The organic, bottom-up approach of repurposing extant social media apps is low cost and efficient for timely implementation to improve patient care. WeChat’s international user base enables medical staff to access widespread professional networks across geographic, administrative, and economic barriers, with potential to reduce health disparities in low-resource settings.

## Introduction

### Background

Health care facilities in low-and-middle-income countries (LMICs) are continually confronted by the public health conundrum of maintaining and upgrading the skills of medical professionals while concomitantly keeping operating costs manageable. The main problem plaguing staff working in LMIC health facilities is that clinicians, technicians, and researchers have limited access to the most up-to-date facilities and knowledge for completing their work. Other difficulties include the need to collectively train the complement hospital staff in complex new procedures, to provide timely analyses of biological specimens for diagnosis, to keep staff abreast of latest developments in trends and clinical procedures and symptoms, and to conduct case reporting for emergent illnesses and update surveillance reports for coordinated international epidemiological efforts. For medical facilities in high-income countries (HICs), a related problem arises in the difficulties associated with the time and resources to formally train clinicians to deliver professional patient services in new online and social media formats [1].

One of the innovative approaches to dealing with these problems is that medical professionals have organically implemented a bottom-up solution by using existing popular apps to devise a mobile health (mHealth) approach [2-9]. Based on publicly available social media technology, the solutions of medical professionals repurposing extant social media application functions are both low cost and efficient in timely implementation. For example, medical doctors use functions within social networking sites (SNSs) to better communicate with each other [4,7,8], and as is the case with WeChat Groups, to coordinate mHealth solutions with medical colleagues to offer around-the-clock question-and-answer support for recently discharged patients with COVID-19 [5]. Since the advent of mHealth into medicine and patient care as early as the mid-1990s [10], and widespread adaption in the 2000s [11,12], social media and internet-based networking apps have demonstrated their usefulness for improving health management systems and coordination of resources for patients and doctors across a range of health care process and institutional settings in LMICs and HICs [10-16].

Recently, the applicability of social media–based tools is becoming more targeted toward application to overcome challenges of limited location-based resources and to improve speediness of information sharing for health care procedures. In addressing these emerging areas of innovation and medical development, there are calls by the international research community for more in-depth [4,17] and qualitative evaluation of the mechanisms of adaption [10,13,14,18,19]. Furthermore, in light of recent pandemics, and national implementation of pandemic-fighting measures such as lockdowns in China and in other countries, these applications are even more critical in ensuring the quality and availability of patient-centered care across diverse socioeconomic settings [20]. In the age of swift-moving global pandemic pressures, the increased burden and mobility restrictions on essential workforce mean that such low-cost, quick, and highly adaptable functionalities become even more important in implementing timely medical assessments and treatment in LMICs and HICs. In fact, in the early days of the COVID-19 pandemic, overseas Chinese in Europe used WeChat Groups to access medical care and consultations from doctors in China [21].

This article contributes to public health discourse by describing the ways that medical professionals use WeChat Groups, an internal function of WeChat app, to overcome barriers in cost, time, physical, and bureaucratic restrictions toward improving patient treatment, diagnosis, training, and information sharing and reporting across geographic and institutional boundaries. The impact of these bottom-up innovative adaptions have resulted in improved diagnoses, consultations, and wider circulation of emerging case reporting information both within and outside the physical territory of China for public dissemination to the global medical community. The implications of these findings are pertinent to resource management in fighting pandemics, such as COVID-19 [21], and to coordinating resources speedily and efficaciously across geographic, administrative, and bureaucratic barriers in LMICs and HICs [22].

In China, the predominant social media app is WeChat, boasting the greatest number of users [23,24], with over 1.2 billion regular monthly users at the start of 2020 [25]. As of 2021, WeChat has over 1.25 billion monthly active users internationally, a growth of 500 million users [26]. Along with Facebook, YouTube, WhatsApp, Facebook Messenger, and Instagram, WeChat is among the top most popular social networking apps globally [27,28]. While Facebook and WhatsApp have the greatest presence internationally, a 2013-2014 consumer behavior study of 170,000 internet users in 32 countries, by Global Web Index (GWI), reported that WeChat is growing faster than rival social media apps in many regions, including in the Americas, that is, Canada (134%), United States (547%), Mexico (2502%), Brazil (1108%), Argentina (835%); in Europe and the Middle East, that is, the United Kingdom (923%), Sweden (152%), Germany (667%), France (778%), Spain (666%), Italy (6587%), Turkey (708%), Saudi Arabia (320%), United Arab Emirates (2716%); and in the Asia-Pacific region, that is, India (1774%), Taiwan (1927%), Singapore (503%), Malaysia (1332%), Indonesia (1094%), the Philippines (2820%), Thailand (191%), and Australia (347%) [29]. WeChat was also the fastest growing app in South Africa (4084%), and the second fastest in Russia (331%), South Korea (259%), and Vietnam (204%) [29]. In fact, WeChat’s platform and success serve as South Africa’s model for extending technology infrastructure and integrating other countries into one social networking system in Africa [30].

At the start of 2014, WeChat had garnered a strong user base, capturing significant portions of the social media apps market in Malaysia (33%), Hong Kong (32%), India (21%), and Indonesia (18%) [29]. By the end of the same year, WeChat solidified its position, with its user base representing 39% in the Asia-Pacific region and 23% globally of GWI study participants [31]. In 2015, there were 697 million, with over 70 million located outside of China [32], with user interphase support available in over 20 languages, including simplified and traditional Chinese characters, English, Indonesian, Spanish, Portuguese, Turkish, Malaysian, Japanese, Korean, Polish, Italian, Thai, Vietnamese, and Russian [32]. Extending its reach beyond regional neighbors, WeChat is reportedly used on a regular basis in Europe—Russia (1%), Finland (1%), France (2%), Germany (2%), Spain (2%), the United Kingdom (2%), Netherlands (3%), and Italy (3%)—and in the United States (3%), Canada (6%), Brazil (2%), and Australia (5%), according to Statistica’s Global Consumer Survey 2020 [33]. By 2021, WeChat became the most popular mobile messenger app in Asia [34].

WeChat is a popular app with a global reach, and its user base and influence as a social media app are growing. WeChat’s reach among China’s global diaspora is undoubtedly significant and serves as a vital means to maintain international connections, despite the controversial bans in the United States and state-sponsored censorship in China [35,36]. Studies of its comparative impact on people’s international information-sharing behaviors, vis-à-vis other prominent social media apps, are underway [37]. Despite its growing influence, the application and adaption of WeChat among health care professionals, along with the implementation of health care innovations, have received less attention. This study contributes to elucidating these processes. As has been illustrated from the start of and throughout the COVID-19 pandemic, connections on social networks have been invaluable for sharing key medical and health care information in a timely manner. Across HICs and LMICs, clinicians are increasingly turning to trusted professional groups on social media to disseminate timely health information across international borders. For example, UK doctors identified a rare blood clot condition for the AstraZeneca COVID-19 vaccination, then turned to WhatsApp to begin sharing symptoms, diagnosis, and treatments, posting daily to 500 colleagues located around the world [2]. WeChat’s popularity and expansive applications for diverse health care settings have grown greatly since its domestic launch in 2011. This paper contributes to the discussion of how the social media app WeChat contributes to technology transfers in health care innovations among medical and health care professions, particularly for patient-centered care.

WeChat has been used in medical and health care settings to improve patient care in China. Clinicians, health-oriented businesses, medical schools, hospitals, and public health infrastructure have adapted it to facilitate scores of innovative solutions to health care management and patient access [15,23,38-40]. As one of the most prominent and inexpensive apps, it has been adapted institutionally in medical technologies in internet (desktop) and social media (mobile-based) platforms for organizing and coordinating patient care. Among hospitals and health care facilities, WeChat has been adapted by administration in several forms, including a WeChat Media (Guanzhonghao) [38], patient registrations and payment (guahao) [41], and appointments with doctors over smartphone consultation and follow-up (dianhua menzhen). Initial costs for facilities include technology support for establishing the pages, in-house technical support to calibrate internal operating systems to online user interfaces, and supplement compensation for doctors and support medical staff who participate in WeChat initiatives (e-consultations) in addition to their regular clinical duties. The origins and general functionalities of the early adaption of WeChat as a mobile app are described in-depth elsewhere [42]. Reports in English-language medical journals further describe WeChat’s versatility and the resulting tangible improvements for a variety of users. Notable examples of the innovative application of WeChat by doctors include their forays to implement pilot randomized controlled trials and feasibility studies [42,43] and to improve patient comprehension of treatment and services by increasing patient-doctor communication [44]. Hospitals have used WeChat to improve patient registration, reduce wait times, and increase timely access of patients to medical staff and health services [41]. Private health care support companies improved the monitoring and collection of personal health data and linkage of health behaviors to medical records and health information [15,16,39]. More recently, WeChat is being incorporated into medical training—implemented in medical and dental schools to organize and train students [45], with a focus on problem-based learning [46,47]; for internship coordination and externship practicums [46]; and to improve the quality of connections between students and their medical instructors for more detailed field evaluation performance assessments [48]. WeChat has also been adapted to indicate health status for patients with COVID-19 and for monitoring public health of the general population [20,22,40].

Despite the institutional applications implemented by medical schools and hospitals, arguably the most exciting development is the emerging bottom-up approach by medical professionals themselves to expedite improvement of services, resource coordination, and information sharing for research purposes. Even before the global push to pitch in for coordinated care on international initiatives to combat COVID-19, medical professionals organically used WeChat Groups to self-coordinate scarce resources, treatment, diagnosis, training, and information sharing of pertinent medical reporting, beyond the boundaries of the traditional hospital. Specifically, doctors, nurses, clinicians, and laboratory technicians and biomedical researchers collectively use self-invited colleague groups via WeChat Groups—unbounded by geography, country, or hospital affiliation. There are private groups and public groups [49-51]. This article describes the private groups created by health care professionals, where entry and information access is gated (ie, only members of the group are able to post and share information with each other). Depending on the number of participants in the group, parameters allow for between 3 and 500 members [50]. Entry is permitted primarily in 1 of 2 ways: invitations are extended by either the group moderator, or by a current group member, to a new potential member [49-51]. The most well-publicized example of WeChat Groups was the international description of how Li Wenliang, an ophthalmologist in Wuhan, announced to his fellow clinical doctors from his medical school alumni group of the presentation of SARS-like symptoms in the early days of the COVID-19 pandemic [52]. Although this disclosure received great attention on the international media as a shocking introduction into the world of WeChat Groups, long-term WeChat users were already familiar with the “Groups” function of the popular mobile app. It is a means of staying in touch and sharing photos with friends across great physical distances and international borders.

In this vein, this article contributes to this growing body of the use of social media in innovative health care solutions by describing the pathways and mechanisms adapted by health care professionals in China to improve diagnoses, consultation, training, and information sharing using WeChat Groups (weixin quan). Specifically, they use a function known as WeChat Groups to create gated groups among their personal network of medical professionals. By doing so, individual medical professionals improve their professional skills and extend institutional capacities of their primary health care facilities by expanding their access to knowledge and technologies beyond their immediate work environments. Using WeChat, clinicians, laboratory technicians, and health care researchers can access new reports, diagnosis, and improve their medical training.

### Objectives

The primary aim of this study was to describe the bottom-up approach adapted by medical professionals across their diverse physical and virtual networks to collectively overcome resource shortages. To accomplish this aim, data collection was focused on delineating the conditions, circumstances, and venues used by medical professionals to connect with other professionals, physically and virtually, before they can begin to ask for and share resources across geographically diverse networks. The secondary aim was to delineate the pathways, procedures, and resource and information-sharing behaviors adapted by medical professionals during the process of repurposing the diverse networks for tasks targeting improvement in patient-centered care. To accomplish this aim, data collection was focused on delineating the specific hospital and health facilities tasks that medical professionals sought assistance for from their social media groups. Specifically, to identify the health care procedures and protocol that medical professions completed by repurposing an international publicly available social media app, to request and share resources to improve patience care.

## Methods

### Setting and Participants

For this project, data collection of ethnographic observations, interviews, and digital ethnography of WeChat person-to-person messaging and WeChat Groups communications occurred from November 2018 to November 2019. Participant observations, qualitative interviews, and digital ethnography of clinical doctors, laboratory technicians, nurses, and medical researchers were based in hospitals/medical facilities in 5 major metropolitan areas (Guangzhou, Shenzhen, Dongguan, Huizhou, and Qingyuan) across the Greater Bay Area/Pearl River Delta of south China. Health facilities covered specializations in dermatology, surgery, chronic illnesses, clinics for treating sexually transmitted illnesses and diseases, general medicine, and oncology.

### Approach

An institutional-level approval for site-based activities was obtained from department/facility administrative leaders. Written and digital copies of project description and informed consent were provided in English and Mandarin. Informed consent for participants was obtained verbally in person and digitally on WeChat. Digital ethnographies of WeChat Groups were compiled in both Chinese and English. Face-to-face interviews and on-site participant observations were conducted in Cantonese, Mandarin, and English.

### Study Inclusion and Exclusion Criteria

Inclusion into the study was determined as follows: (1) employed as a medical/health care professional with experience working in hospital/medical settings or having attended schooling for medical, nursing, pharmacy, laboratory, or health sciences; (2) had prior/current exposure to or application of international transfers of technology, in the form of knowledge, skills, abilities, work competencies, or training; (3) had acquired these knowledge, skills, abilities, and competencies through formal study abroad, overseas training or conference participation, domestic training from international partners, domestic engagement with international working/scholarly partnerships, and training materials; and (4) uses internet-based technologies (ie, social media) to communicate for professional/work interactions. Exclusion from the study was determined as follows: (1) not a trained medical or health care professional despite currently working or formerly having worked in hospital or medical settings; (2) was not exposed to, does not work, or does not follow or use any international guidelines, equipment, reading or training materials, or technologies; (3) not currently nor has ever engaged in any medical or health professional social networks with international connections; or (4) did not use internet-based technologies in any work-related tasks or functions.

Construction of the frame for participant enrollment was a multistep process. To ensure a robust framework for enrollment into the study, prospective participants were contacted through 3 categories of network connection types: formal networks, semiformal networks, and informal networks. In interactions across these 3 network categories, informants were asked if they could help identify potential participants throughout the Greater Bay Area region to encompass major urban centers, semirural peripheries, as well as rural areas. Further, informants were asked to help ensure diversity of participant background by identifying and introducing contacts who have experience working in each of China’s 3-tier system of hospital and medical facilities [53], defined as primary level (village/community/township; <100 beds), secondary level (district/county; 100-500 beds), and tertiary-level (comprehensive-city/provincial/national; 500+ beds). At each meeting with informants, and after the conclusion of each participant interview, informants and participants would be asked if they had colleagues or acquaintances to recommend for the study. If the response was positive, then they would be asked if they could ask their contacts, and send along a copy of the researcher’s WeChat business card, curriculum vitae, and bilingual copy of the informed consent.

Formal networks were based on institutional connections made through requests at medical establishments, such as medical schools, hospital and hospital administrative systems, medical associations, and government/public health administrative agencies. Major medical establishments in the Greater Bay Area (consisting of Guangdong Province in China, Hong Kong, and Macau) were contacted to obtain institutional support for the study. Support meant that the establishment would provide a liaison to help contact potential participants employed as staff at their home institution, or those that were alumni or active association members (ie, doctors, nurses, pharmacists, laboratory technicians, medical researchers, public health administrators with medical training). During the prospective enrollment process, local leaders and the study liaisons confirmed to potential participants that there would be no adverse impact for participating in the study, and that no data would be shared with their work units or network institution. The approval process involved firstly sharing a copy of the study informed consent, written in English and in Chinese, a digital copy of the researcher’s curriculum vitae in English, and a digital copy of the researcher’s bilingual business card via WeChat. Next, upon approval from the medical establishment, the institutional liaison assigned to formally support the study would establish appropriate departmental clearance and make connections with potential participants. Finally, institutional support for the study at these sites meant that participants could sit for an interview during normal work hours and at work facilities. At these sites, the institutional liaison conformed to the study inclusion criteria in identifying potential participants. Specifically, inclusion into the study from these formal connections included having worked in their hospital/medical facility as a medical or health professional as current full-time employees. Potential participants were then invited to face-to-face interviews individually on a 1-on-1 basis, sitting for an interview and sharing their WeChat contact information in an enclosed, secure, physical location separate from other colleagues. At the start of the interview, participants were asked if they willingly want to participate in the study, and if they agreed, were presented with a hard copy of the informed consent. Each participant was asked a series of inclusion questions to verify eligibility.

Semiformal networks were connections made from study participants (originally from the formal networks) who were willing to serve as informants to make introductions to prospective participants from their own social networks. These prospective participants would be personal or professional network contacts, originally met through work, school alumni associations, or some type of professional capacity. They do not have current formal institutional (reporting or administrative) relationships with informants, to ensure that the interviews are not compelled. As semiformal networks did not require institutional support, informants who were already familiar with the interview and participant observation processes assessed which members of their social networks were trained medical and health professionals who fulfilled the inclusion criteria. Serving as informants, these participants would ask their contacts individually. If the prospective agreed to being interviewed, the informant would then send each party a contact card invitation within WeChat. After a prospective participant accepted the invitation to connect on WeChat, a copy of the informed consent and the bilingual business card would be sent. Interviews were then conducted either in-person at a neutral public location, online via WeChat, or in-person at the workplace of the participant.

Informal networks were connections made without the assistance of formal and semiformal informants, primarily through 1-on-1 engagement and interactions at health-oriented conferences, conventions, meetings, and networking events. These events were advertised to or promoted by informants from the formal and semiformal networks, but there were no direct links or contacts from them for connections at these venues. These venues were recommended by informants as professional events they themselves would normally attend, or be invited to, to connect in a professional capacity. At these events, potential participants from well-known health facilities (with operational presence in the Greater Bay Area) were approached, and asked if their organization as an institution, or health professionals from their organization individually, would be interested in participating in the study. At the initial meeting, prospective participants were presented with a hardcopy of the informed consent, and given a hardcopy of the bilingual business card. If they were then interested in participating, they would be asked the series of inclusion questions to verify eligibility. Prospective participants could decide when and where to conduct the face-to-face interview.

During the interview, participants were asked about their use of online social networking, WeChat usage, if they could describe or demonstrate some of the ways they use WeChat and their smartphones to support their medical and health care–related work tasks, and if they would be willing to connect on WeChat for digital communication and continued participation. When individual 1-on-1 connections were then made on WeChat, participants were sent an electronic copy of the study institutional review board approval. To observe participants using WeChat Groups as part of the digital ethnography, throughout the study, participants from formal, semiformal, and informal networks would share invitations with specific WeChat Groups that they were members of, making introductions to the other WeChat Groups participants. The researcher would then introduce the study, and share a copy of the informed consent and bilingual business card.

### Data Management and Analysis

Qualitative data comprising field notes were typed in English and interviews were transcribed into Mandarin and English. These data were inputted and managed in NVivo. Thematic analysis [54,55] was supported by a deductive approach to the data and coded within NVivo. The main themes from interviews and descriptive observations from field notes and interviews were reported across the occupational specialty of participants.

### Ethical Approval

This study was conducted in accordance with General Data Protection Regulation (GDPR) and approved by the University of Oslo Ethics Committee as part of IKOS grant number 275002 (2018).

## Results

### Overview

Interviews, digital ethnographies, and observations were conducted for 58 people (36 men, 22 women) and were included in the final analysis. Participants included doctors (clinicians, surgeons, and medical researchers), nurses, laboratory technicians, hospital pharmacists, and public health administrators (Table 1).

**Table 1:** Participant demographics, Guangdong, China, November 2018-2019 (N=58).

Characteristic			Participants
**Gender, n (%)**			
	Male	36 (62)	
	Female	22 (38)	
**Medical profession, n (%)**			
	Doctors^a^	39 (67)	
	Nurses	3 (5)	
	Laboratory technicians	7 (12)	
	Pharmacists	2 (3)	
	Public health administrators	7 (12)	

^a^Includes clinician, surgeons, and researchers.

Field sites included 24 health care facilities from Guangzhou (n=19, 79.2%), Dongguan (n=2, 8.3%), Shenzhen (n=1, 4.2%), Qingyuan (n=1, 4.2%), and Huizhou (n=1, 4.2%). Digital ethnographies were collected from online sources for each site. On-site visits were performed for 13 of the 24 (54.2%) health care facilities for extended interviews and in-person observations.

### Constructs and Themes on WeChat Groups to Improve Patient-Centered Care

Analysis of the digital ethnography, field observations, and qualitative interviews revealed 4 construct sets grouped into 11 main themes for categorizing observations from site visits and participant interviews. Results are summarized in Table 2.

Overall, the bottom-up–driven creation of WeChat Groups among medical professionals’ networks facilitated participants’ skill development and knowledge broadening, leading to the improved ability to provide patient care and enhance specialization in their respective subfields. Specific pathways and mechanisms include establishing and utilizing connections in a widening network, enhanced opportunities in training and continuing education, improved access to knowledge base and equipment for diagnostics and procedures, and higher quality contact and linkage to patient histories. These processes collectively improve patient care via diagnosis, consultations, symptom/history reporting, and treatments, thereby improving quality and frequency of information sharing across diverse settings and geographic boundaries.

**Table 2:** Constructs and themes for use of WeChat Groups to improve patient care, medical training, and case reporting in health care settings in China, 2018-2019.

Construct	Theme
Establishing and Utilizing Connections in a Widening Network (Construct 1)	Physical mobility improved knowledge of resource location, variety of activities, patient subgroup, disease concentrations (Theme 1) Geographic coverage and bridging administrative boundaries (Theme 2) WeChat Groups connectivity strengthened connections for support and opened up opportunities for information sharing, training venues, and continuing education (Theme 3)
Training and Continuing Education (Construct 2)	Reading groups to improve skills up-training and continuing education (Theme 4) Access to paywall articles and books outside of China (Theme 5) Reducing barriers to differential access to international conference reporting, surveillance data, and medical treatment guidelines (Theme 6)
Improved Access to Knowledge Base and Equipment for Diagnostics and Procedures (Construct 3)	Tapping the hive mind for knowledge and resources for diagnostic interpretation (Theme 7) Improved communication, better access to scarce resources located far away, group-mind/collective diagnosis (Theme 8) Research coordination and manuscript preparation (Theme 9)
Higher-Quality Patient Contact and Linkage to Patient Histories (Construct 4)	Local-to-provincial consultation support (Theme 10) Better initial and follow-up contact and care for patients (Theme 11)

### Construct 1: Establishing and Utilizing Connections in a Widening Network

#### Theme 1: Physical Mobility Improved Knowledge of Resource Location, Variety of Activities, Patient Subgroup, Disease Concentrations

While most participants were based in health facilities in Guangzhou, there was in fact a great deal of mobility among participants. As part of their clinical work, surveillance reporting, and continuing education, medical professionals routinely engaged in intraprovincial travel within Guangdong Province, and across the border to Hong Kong, Macau, and Taiwan. There were several annual medical conventions that were held in Shenzhen and Zhuhai, whereby participants would intermingle and engage with their networks. With frequent contact from these annual international, national, and provincial conventions, along with annual and quarterly professional training meetings, participants could meet other medical professionals in person, learn about facilities and resources of their colleagues, and maintain contact to ask for support in their expanding WeChat Groups connections.

#### Theme 2: Geographic Coverage and Bridging Administrative Boundaries

Participants described that their professional networks on WeChat Groups extended beyond their home health care facility. WeChat Groups connections were created based on similarities in alma mater via informal alumni networks, professional colleagues in study abroad/medical internship programs, continuing education/professional specializations, formal affiliate hospital programs, and surveillance-reporting administrative structures. Hence, these WeChat Groups connections map onto professional networks that geographically span neighboring hospitals, across city-province connections, extending into the South China region to Hong Kong, Macau, and Taiwan, and reaching into international major research hubs in Japan, the United States, and Europe.

#### Theme 3: WeChat Groups Connectivity Strengthened Connections for Support and Opened up Opportunities for Information Sharing, Training Venues, and Continuing Education

After meeting in person, participants used these WeChat Groups connections to stay in touch digitally on a regular basis for professional support, sharing training event updates and relevant materials, and coordinating site visits with colleagues in other hospitals and health facilities. This was particularly the case for locations that specialize in certain treatments, procedures, operations, or patient populations. Participants located in more remote locations (away from Guangzhou) learned about the implementation of new technologies and techniques, and asked counterparts to share more information for local skills upgrading and training. Once part of an invited private group within WeChat Groups, it was possible to submit requests for assistance to other people in WeChat Groups that one may not have met in person before.

### Construct 2: Training and Continuing Education

#### Theme 4: Reading Groups to Improve Skills Up-Training and Continuing Education

One of the more novel ways of using WeChat Groups observed in the field is the establishment of thematic reading groups (for English-language materials). Medical professionals, particularly doctors, would identify subject topics they collectively want to learn more about. Then they would share relevant research articles, book chapters, or diagnostic guidelines. On WeChat Groups, common problems in digesting the text were discussed, and supplemental materials would be shared. The materials most often shared among WeChat Groups connections established as reading groups were general public access medical guidelines published online, PDF files of biomedical articles from online digital research repositories (eg, PubMed), PDF files of paywall-access materials obtained via an institutional account from a WeChat Groups member, digital copies of book chapters or medical textbooks shared by publishers with WeChat Groups members, and professional conference presentations content. Oftentimes, the reading groups would meet in person to collectively read the text to identify new terminology, procedures, and cutting-edge techniques and procedures applicable to clinical duties, biological sampling protocols, and out-patient surgical procedures.

#### Theme 5: Access to Paywall Articles and Books Outside of China

In recent years, health care facilities in China have gained greater access to medical databases and peer-reviewed journals. The push for open publishing and wider access to PubMed have made it much easier for medical professionals to access information on symptoms and disease development and progression. However, there are still many articles that are behind pricy subscription paywalls, and books that are too expensive for the acquisition budgets of smaller hospitals. Hence, participants turn to different connections in separate WeChat Groups, asking if group members have electronic copies that they can share directly. Participants in government institutions and in primary- and secondary-level rural-based hospitals acknowledge that colleagues in academic settings tend to have better access to these key resources. Within WeChat Groups, members with access to institutional accounts would obtain PDFs by searching for and accessing paywall articles, digital book chapters, and digitized books and textbooks. As these institutional accounts fulfill copyright laws and regulations, the subsequent limited amount of scholarly sharing is generally thought of as covered by the fair use exception for sharing these materials for educational and scholarly purposes.

#### Theme 6: Reducing Barriers to Differential Access to International Conference Reporting, Surveillance Data, and Medical Treatment Guidelines

Similarly, there seems to be differential access to international surveillance reports and reporting updates to international benchmark guidebooks. Participants indicated that doctors and technicians involved in international research collaborations tend to know about and gain access to international reports more readily than clinicians and public health administrators. As many medical professionals in China (and other LMICs) experience financial and bureaucratic constraints on attending international conferences, colleagues from academic institutions and competitive research–implementation collaborators share the newest guidelines, medications, and treatment regimens for patient care via their participation in various WeChat Groups activities.

For doctors, use of WeChat Groups helped improve their efficiency in patient-oriented care processes by facilitating their work in 3 primary ways. First, by using WeChat Groups, doctors could ask for help in locating descriptions of symptoms on articles posted on PubMed. PubMed is a free online resource as part of the US National Library of Medicine, and consists of search-and-retrievable open-access abstracts and links to millions of high-quality peer-reviewed biomedical and life sciences research studies. However, because the interphase is in English, and being unfamiliar with site layout and system navigation, WeChat Groups users may need assistance in acquiring the knowledge and ability to navigate the English language site, to perform content searches and to access the relevant linked articles. Second, WeChat Groups helped doctors, particularly those located in primary and secondary hospitals in rural locations or low-resources settings, access the newest international guidelines on disease symptoms. These doctors are aware that updated content may have been presented at international conferences and national meetings attended by WeChat Groups members with access to greater financial resources. To help them accomplish their diagnostic and clinical work more efficiently by learning about updated guidelines, new clinical biological specimen sampling, and surgical procedures, WeChat Groups members may ask for copies of slides, PDFs, short videos, or presentation papers circulated at these events. Third, in reviewing the guidelines on the WeChat Groups platform, the members would hold discussions on how, where, when, and under which conditions the guidelines can be applied in clinical evaluations of symptoms for patient consultations and laboratory specimens. These mechanisms are now more easily performed via WeChat Groups. Overall, WeChat Groups support more efficient peer-to-peer upskill training of medical professionals for acquiring knowledge and skills delineated in updated guidelines, medications, and treatment regimens for patient consultation and surgical care.

### Construct 3: Improved Access to Knowledge Base and Equipment for Diagnostics and Procedures

#### Theme 7: Tapping the Hive Mind for Knowledge and Resources for Diagnostic Interpretation

It is common practice for medical professionals to take photographs of patients’ ailments, unprocessed biosamples, and processed specimens for health record documentation, surveillance and reporting, and research dissemination purposes. The innovative application by medical professionals is the uploading of these photographs to specialist WeChat Groups to support diagnostics and interpretation.

During analyses, the concept of becoming more efficient in accomplishing health care processes arose from interviews and observations of the health care professionals as they carried out their daily work. They described how their use of WeChat Groups helped them become more efficient at their tasks. Turning to WeChat Groups, members can streamline processes and procedures that previously took a great deal of time, required multiple steps, or were just not feasible before the implementation of bottom-up work-arounds using WeChat Groups. This access to a hive-mind approach is a shift in the ways that health professionals conceptualize their “available resources at hand.” For example, it is customary for doctors at primary facilities (usually based in rural areas with low resources) to refer patients with unresolved ailments up to secondary (at township or city levels) or tertiary facilities (at provincial centers and regional hubs). Because the doctors, nurses, and laboratory technicians are part of a larger, integrated health care infrastructure, they can now use WeChat Groups to readily share pertinent information on symptoms, treatments, and evaluations of laboratory specimens. Previously, these upstream consultations with higher-level doctors may happen at quarterly in-person check-ins. It is now feasible to directly message the connections in WeChat Groups for support in diagnoses and consultations. For particularly difficult cases, it would be feasible to ask for support and help from WeChat Groups members working in facilities with more advanced equipment, either overseas or at national centers. Knowledge of these international linkages and nodes of resources are often shared at conferences during presentations and at large-scale yearly/quarterly training sessions across multiple hospitals in the same health care system. By connecting with members on WeChat Groups, medical professionals based in primary and secondary hospital settings can more quickly treat their local patients. For particularly difficult cases that required higher levels of access to medical diagnostic equipment or diagnosis evaluations, laboratory staff and medical clinicians would request support from WeChat Groups members based overseas for traineeships in medical and research facilities in locations such as the United States, Europe, and Japan. Hence, despite being based in a rural or low-resource hospital, health professionals are better connected to a wider medical knowledge base and diagnostic services in their home country, with increased access to high-quality Chinese- and English-language medical knowledge and diagnostic resources available abroad.

#### Theme 8: Improved Communication, Better Access to Scarce Resources Located Far Away, Group-Mind/Collective Diagnosis

Related to the hive minding of resources, if a health facility lacks specific equipment to provide an in-depth analysis, the photographs and accompanying medical information (deidentified of linkable patient data) are shared with members via WeChat Groups. The result being a hive-mind processing for knowledge base assessment and access to equipment at resource-rich locations. This pathway of accessing resources was described by laboratory technicians, whereby they take photographs of biospecimens, and would share the initial reporting with colleagues based in health facilities with more advanced equipment to obtain diagnostic support. This process could be considered an alternate form of telemedicine. Participants described using this process on WeChat Groups to connect with members located in the United States, asking for assistance to help run diagnostics for patients from health facilities in China.

#### Theme 9: Research Coordination and Manuscript Preparation

An increasingly prevalent concern among medical professionals in China is the growing pressure to produce research concomitant with clinical duties. As promotion and pay become linked to publication and journal prestige in Chinese medical systems, participants began using WeChat Groups to support research coordination and manuscript preparation. Clinically oriented staff and functional departments began to develop resources for sharing literature reviews, guidelines for implementing systematic reviews, sharing results of quantitative and diagnostic analyses, and manuscripts drafts using WeChat Groups.

### Construct 4: Higher-Quality Patient Contact and Linkage to Patient Histories

#### Theme 10: Local-to-Provincial Consultation Support

In China, public hospitals are organized in a tiered hierarchy, with affiliate hospitals at the province, county, and township levels. When dealing with an ailment, many patients tend to try their local hospitals first. However, if the health professionals are unsure of the symptoms, clinical procedures, treatment, or medications, they may contact their colleagues higher-up in their surveillance reporting hierarchy to ask for clarification and assistance.

#### Theme 11: Better Initial and Follow-Up Contact and Care for Patients

Similar to the in-network patient referral process in US health systems, patients in China can be referred to specialists and departments at a higher-level affiliate hospital. This process can be disorienting for patients, so clinicians at local hospitals may contact their doctors in their surveillance reporting unit to ask for help in treating patients with problematic symptoms. In severe cases, the patients will be referred to the county, city, or provincial hospital. When necessary, doctors will coordinate care for patients in this manner, organizing surgeries and special consultations in affiliate units. The progression up this hierarchy is often used of organizing surgeries for patients. This practice is now becoming commonplace for diagnosis and treatment of infectious diseases and sexually transmitted illnesses and diseases. In this study, participants described how if patients are not able to get enough help in remote locations, or lower-tier locations, they go to Guangzhou. Then, it is possible for doctors to coordinate help with diagnostics and referrals and establish history of symptoms and treatment regimes.

## Discussion

### Principal Findings

How does WeChat Groups help medical professionals? In many LMICs, hospitals often lack access to the latest medical information and equipment for treating patients. In devising solutions to this dilemma, doctors and their support staff have come up with innovative ways to utilize online technologies and social media networks to overcome barriers in physical infrastructure. These solutions are beneficial to improving patient-centered care in LMICs and HICs alike.

One of the important findings of this study is that the establishment and agentic utility of a professional network for medical professionals are magnified and widened by using WeChat Groups. In fact, WeChat Groups now serve as a vital pathway for doctors, nurses, technicians, pharmacists, and public health administrators to access resources and share important occupation-based knowledge and procedures across geographic and administrative boundaries. This finding builds on reviews published by JMIR about health professionals using SNSs to build virtual communities and engage in professional communication [4,7-9]. It extends on previous findings by documenting the channels and mechanisms used to adapt SNSs [4], showing how doctors have progressed beyond using SNSs for discussions about medical topics into repurposing apps to better perform health facility–based procedures and protocols [7]. Perhaps most importantly, WeChat Groups support communication across administrative barriers and great geographical distances internationally to improve dissemination of emerging local health crises along trusted member networks. By repurposing SNSs, doctors shared life-saving information on health care procedures to combat COVID-19 [2,5,21,52,56]. To coordinate resources for addressing localized emergencies, it is possible to notify members with differential resources to share them with members working with fewer resources. Willing members share resources and information, overcoming some of the distribution inequities by tapping into richer regions in China and abroad. Participants who have better access to paywall resources or better virtual private network (VPN)–controlled access to international materials can share these resources more easily using WeChat Groups. For example, first during their 1-on-1 interviews participants who reported obtaining information, and later, during participant observations, were observed using WeChat Groups to access World Health Organization (WHO) reports, country-specific Centers for Disease Control and Prevention (CDC) updates, including diagnostic guidelines and reports; conference proceedings from international events; and international diagnostic treatment and medication guidelines for illnesses. They requested and shared these materials collectively from WeChat Groups members and connections dedicated to occupational reading, paper writing, and implementing new international procedures.

Participants described how they use WeChat Groups to share information with each other. Using the sharing functionalities within WeChat Groups, health professionals are exchanging materials in English and Chinese, reflecting the internationalization of medical care and China’s engagement within the international medical community. Collectively, resources shared include open-source journals, industry-specific guidelines and reports, and paywall subscription products that group members access via their institutional credentials to then share with their colleagues.

Within a large-scale health care structure, such as a comprehensive hospital, there may be several important health facilities/subgroups. From the analysis and results, WeChat Groups offer complementary mechanisms to achieve larger health care goals of improved patient care, lower opportunity costs in time and resources, and quicker sharing of pertinent health-related information. For example, hive-mind group–based diagnosis of images and laboratory results (laboratory technicians); sharing articles, medical papers, and reports for symptoms and diseases (doctors and nurses); textbooks and guidelines used for prepping surgeries and operative care (surgeons); newest diagnosis/screening techniques (clinicians and nurses); better access to international conference materials, guidelines, evidence-based surveillance reports (public health administrators); newer drug information; and wider networks to sell and source medications for hospital’s in-house needs (pharmacists). These functions indicate that WeChat Groups not only concretely improve the pathways and mechanism of patient-centered care, but also provide additional opportunities to improve the timetable, budgeting, and strategic operations of health care facilities.

The description of health and medical professionals using WeChat Groups, and finding it offers an “efficient” approach to carrying out procedures, arose from analysis of the interviews and participant observations. During fieldwork and interviews, clinical doctors and health care professionals who work in hospital settings, namely, laboratory staff, described the efficiency as saving time and labor, and reducing steps to completion of procedural steps in patient care. Participants described increasing their efficiency by tapping into the specialist colleagues via WeChat Groups for help in the diagnosis of samples. For doctors, tapping into their professional contacts facilitated their work in 3 primary ways. First, it helped them locate descriptions of symptoms on articles posted on PubMed. Second, it helped them access the newest international guidelines on disease symptoms and on updated content presented at international conferences attended by WeChat Groups members with access to more financial resources. Third, in reviewing the guidelines on the platform, members would hold discussions about how, where, when, and under which conditions the guidelines can be applied in the clinical evaluations of symptoms for patient consultations and laboratory specimens. These 4 key mechanisms, described by and observed among clinicians and laboratory staff, delineate concrete ways in which using WeChat Groups helped them perform necessary health care procedures more effectively. In sum, health care professionals found this approach to be efficient because it helped in reducing the time from first consultation to diagnosis, checking for continued adherence to newly updated diagnostic and treatment guidelines, and obtaining second opinions from other doctors and health care professionals in their networks who logged more experience and continued medical education training via international conferences, national meetings, and overseas traineeships.

Collectively, clinicians, technicians, and researchers have limited access to the most up-to-date facilities and knowledge for their work. The difficulties include the need to train the hospital staff, provide analyses of biological specimens for diagnosis, and keep staff abreast of latest developments in trends and clinical procedures. Overall, medical professionals use WeChat Groups to access information and resources and support their staff’s capacity to share resources across social networks. These functions, both offline and online, are becoming increasing important in the transfer of technologies, skills, and knowledge from HIC into LMIC settings to support equitable global development and improve global health equity. In this vein, clinicians across several disciplines have identified novel ways to utilize WeChat Groups for training of students in multimodal curriculums [46-48]. The solution that organically arose is to use WeChat Groups to effectively pool resources and knowledge from social networks that map beyond the physical limitations of a hospital or single medical facility.

### Limitations

This study demonstrates the pathways of information sharing engaged by medical professionals within and outside of China, but is limited in discerning the strength of these interactions. During the study data collection, participants highlighted the locations and venues where their connections were first made, describing why trust was important for validating linkages, and relating how trust was established for other members in a given professional group established within WeChat Groups. In-person observations, interviews, and digital ethnographies were used to identify the differentiated roles that various members in a group performed. However, one of the limitations of this study is that follow-ups with individual participants were limited, hence information on why individual connections within a group were made is reliant on interview information collected during initial entry into the study. Observations of which individuals were tapped for various types of information, what kind of resources were used to obtain the information, or how the data shared in the online setting of social media transcended into improvements in medical transcription of patient records or offline patient cares services are limited to WeChat Groups chat functions only. Further studies into the next steps of patient care could help improve knowledge of how medical professionals implement tailored services for patients across LMIC settings across geographic barriers.

Another limitation of the study is that there was minimal documentation, interview questions, and participant observations of the technological deficiencies in the functions of WeChat, or the impact of these technological issues on the dynamics of WeChat Groups. For example, in the wider literature, issues such as restrictions on the maximize size of a file upload may have imposed size limitations on information sharing for large files, such as videos clips of presentations. Another technological difficulty was that images and files that are not immediately downloaded and saved onto the hard drive of the smartphone or onto a synced-up account on a computer could be deemed “unavailable” after the regularly scheduled purge. At times, when certain websites or links could not go through due to censorship algorithms, WeChat Groups members would take screenshots of the websites or content, then share the screenshots as a series of photograph images. Overall, these technological deficiencies in WeChat and WeChat Groups had minimal impact, and were readily dealt with in the data collection process because WeChat users in China are accustomed to creative solutions in circumventing restrictions [56]. As part of the digital ethnography data collection process, when technical issues arose on WeChat or within WeChat Groups, the impacted study participants were directly contacted on WeChat via text message or WeChat call on a 1-on-1 basis, by phone call or email, and alternative methods for sharing large files, for circumventing censorship algorithms, or for resharing the “unavailable” files again, and thus succeeding in completing data collection. Future research could delve into the technological deficiencies and the bottom-up work-arounds developed by health professionals to overcome restrictions in information sharing.

### Conclusion

Implementation of new digital technologies in medicine is important in maintaining excellence and innovation in health care across low-, middle-, and high-income settings. Publicly available popular social media apps can help save time and reduce costly staff upskills training, offering an increasingly invaluable option for continuing medical education and eHealth around the world [13]. In the wake of the global pandemic caused by COVID-19, health professionals are increasingly turning to social media messaging apps to share life-saving diagnosis and treatment information across closed international borders [2]. This paper illuminates emergent, ethnographic, and qualitative methodologies to investigate the linkages, actors, and pathways in the sharing of information and resources across spatial differentials. Moreover, these results can help inform feasibility and scalability studies [13,18] by providing evidence-based development of appropriate metrics for future quantitative assessments and evaluations of social media implementation programs. Furthermore, this paper describes the socioeconomic and geospatial disparities experienced in LMICs, and the innovative, bottom-up approaches and concomitant pathways and mechanisms devised by medical professionals to overcome these barriers. These processes of information sharing and internal resource redistribution can improve health equity in international patient-centered care. This paper contributes to efforts in implementing transfers of technologies, knowledge, skills, and abilities to support equitable global development and global health equity in low-resource settings.

## Abbreviations

CDC: Centers for Disease Control and Prevention
GDPR: General Data Protection Regulation
HIC: high-income country
LMIC: low-and-middle-income country
mHealth: mobile health
SNS: social networking site
VPN: virtual private network
WHO: World Health Organization
